# The Effect of Treatment With Aminoguanidine, an Advanced Glycation End Product Inhibitor, on Streptozotocin-Induced Diabetic Rats and Its Effects on Physiological and Renal Functions

**DOI:** 10.7759/cureus.42426

**Published:** 2023-07-25

**Authors:** Ram Mukka R Jogula, Anupama T Row, Athar H Siddiqui

**Affiliations:** 1 School of Medical Sciences, University of Hyderabad, Hyderabad, IND; 2 Department of Pathology, University Health Center, University of Hyderabad, Hyderabad, IND

**Keywords:** renin-angiotensin system, aminoguanidine, advanced glycation end products, renal functions, angiotensin ii receptors, diabetes

## Abstract

Background/aim: Diabetes is a multifactorial syndrome that affects the functioning of the renin-angiotensin system (RAS). The role of advanced glycation end products (AGEs) in diabetes is well known. In the present study, we hypothesized that the prevention of AGE accumulation or abrogation of AGE synthesis using an AGE inhibitor, aminoguanidine (AG), in streptozotocin (STZ)-induced diabetic animal models would affect the progression of diabetes and its related complications. We determined the effects of aminoguanidine (AG), an AGE inhibitor, in STZ-induced diabetic rats by determining various indices of RAS and renal functions. Additionally, we also investigated the effect of the drug, AG, on various hemodynamic and physiological functions in the body of the animals.

Methods: Male Sprague Dawley rats weighing 200-250 g were assigned to four groups (n = 4-6): Vehicle, Vehicle+AG, STZ-induced, and STZ-induced+AG rats. Type 1 diabetes was induced by a single intraperitoneal (IP) injection of streptozotocin (55 mg/kg) dissolved in sodium citrate buffer. The control groups (Vehicle) were injected with buffer. The blood glucose levels were measured after 48 hours, and animals with blood glucose levels > 300 mg/dL were included in the study. Blood glucose levels in the vehicle rats were also determined to ensure non-diabetic conditions. After confirmation, AG was administrated at a dose of 1 g/L in drinking water for two weeks. Urine was collected to measure the glomerular filtration rate (GFR), and the immune reactivity for AT_1_ and AT_2_ proteins was analyzed by immunoblotting. Data were expressed as mean ± standard error of the mean (SEM), and a p-value < 0.05 was considered statistically significant.

Results: Diabetic rats had a significant drop in body weight, accompanied by increased food and water consumption. The diabetic rats exhibited significantly increased urine flow and GFR. These phenotypes were significantly or considerately reversed by AG treatment in the STZ+AG-treated diabetic rats. Aminoguanidine prevented the increase in blood sugar levels compared to STZ-induced diabetic rats alone (295.9 ± 50.69 versus 462.3 ± 18.6 mg/dL (p < 0.05)). However, it did not affect the glomerular filtration rate (GFR) and glomerular damage, as assessed by the renal histopathological studies. The STZ-induced diabetic rats had an increased sodium excretion (3.24 ± 0.40 mmol) and significantly increased expression of the AT_2_ receptor and that of the AT_1_ receptor, which was slightly reversed by the treatment with AG. Treatment with AG decreased sodium excretion (2.12 ± 0.63, as compared to the diabetic rats). These rats also had modestly decreased expression of the AT_2_ receptor (0.99 ± 0.07 versus 1.12 ± 0.08, as compared to the STZ-induced diabetic rats), while the AT_1_ receptors showed a slight increase in the STZ+AG-treated rats compared to the STZ-induced diabetic rats (1.1 ± 0.19 versus 1.08 ± 0.12).

Conclusion: This study highlights the action of the drug AG in not exacerbating any damage in diabetic rats. Employing AG as a pharmacological intervention to prevent an increase in blood sugar adds a new dimension to controlling increased blood sugar and preventing diabetic complications. The employability and pharmacological intervention of the drug AG, in diabetes, therefore, need a renewed and further investigation.

## Introduction

Diabetes is a multifactorial disease caused by a lack of insulin or insulin resistance [1]. It is mainly characterized by increased blood sugar levels. Epidemiological studies have revealed that more than one in 10 adults are now living with diabetes, and the global diabetic population is estimated to reach 637 million by 2030 [2].

The altered physiological state in diabetes, especially with regard to fluid homeostasis, as a consequence of polydipsia and polyuria, activates the factors that are critical in maintaining the water and salt balance, especially the sodium levels, in the body. The factors that regulate the ion and water balance mainly include the renin-angiotensin system (RAS) [3].

The components of RAS mainly comprise hormones (angiotensinogen, angiotensin II (Ang II), and aldosterone), enzymes (renin and angiotensin-converting enzyme), and receptors (AT_1_ and AT_2_). Angiotensin II (Ang II), the principal effector hormone of the RAS, plays a very important role in the manifestation of hypertension and in the progression of renal diseases [4]. The receptors for Ang II are abundantly expressed in the kidney, heart, eye, and brain and play an important role in vasoconstriction, activation of the sympathetic nervous system, and regulation of the sodium/fluid balance, mainly at the renal axis [4]. Abnormalities in the RAS, mainly due to the changes in the plasma renin activity (PRA), angiotensin II, and their receptors, lead to dysregulation of RAS causing hypertension under diabetic conditions [5,6].

Previous studies have shown that enhanced AT_1_ receptor function has been implicated in various cardiovascular and metabolic diseases such as diabetes and obesity [7]. In vivo [8,9] and in vitro [10] studies have shown increased renal AT_1_ protein expression and function in sodium reabsorption through the activation of the Na+/K+ ATPase pump [11]. A dual response of Ang II receptors, in terms of maintaining sodium levels in the body, has been reported in hypertensive rats and suggested that it is a critical determinant in regulating blood pressure [12]. Contrarily, reduced renal AT_1_ expression has also been reported in diabetes, which may contribute to abnormalities in volume regulation and acid/base balance [10,13]. At the same time, the function of the AT_2_ receptor is least known. There is evidence of the distribution of AT_2_ receptors in several tissues and altered function under pathological conditions [6]. A study conducted on obese Zucker rats revealed that increased expression of the AT_2_ receptor in the kidney promotes natriuresis/diuresis [14]. Similarly, another study showed increased expression of the tubular AT_2_ receptors and its role in natriuresis via the guanosine 3',5'-cyclic monophosphate (cGMP) pathway in streptozotocin (STZ)-induced diabetic rats [15].

In this regard, Ang II receptor blockers (ARBs) and angiotensin-converting enzyme inhibitors (ACEIs) are clinically used as the first line of therapeutic agents for the treatment of hypertension in diabetes [5,16]. However, the incidence of diabetes complications is increasing at an alarming rate, but the exact cellular or molecular basis of these complications has not yet been fully elucidated. One of these events is the formation of advanced glycation end products (AGEs) by non-enzymatic reaction [17]. Earlier in vitro studies have shown that glycation within the cell can occur under elevated glucose conditions, leading to a loss of glucose responsiveness that can be improved by inhibitors of glycation [18]. The formation of AGEs does play a role in glucose toxicity [18] involved in irreversible crosslinking with extracellular matrix proteins [17] and the inhibition of the β-cell function such as insulin release and biosynthesis [19]. In addition, studies reported that the binding of AGEs to its receptor RAGE contributes to oxidative stress, inflammation, and metabolic and structural defects in diabetic renal/vascular disease [20].

Existing in vitro and in vivo data suggested that the ACEIs and Ang II receptor antagonists were reported to decrease the formation of AGEs and prevent oxidative stress and inflammation under diabetic nephropathy [21,22]. The blockade of the RAS by the AT_1_ receptor antagonist, losartan, attenuated the accumulation of serum AGEs in a non-diabetic model of renal disease [23]. Therefore, treatment with agents such as the AGE inhibitor may be of advantage in retarding the deterioration of β-cell function in diabetes.

Hence, we hypothesized that aminoguanidine (AG), an AGEI, if administered before the onset of diabetes, could prevent or ameliorate the complications associated with diabetes by preventing the generation of AGEs and may halt the progress of diabetic complications. The novelty of this study focused mainly on two aspects, arising out of the background and the rethink about the role of AGEs in diabetes and hypertension under diabetes. While a convincing role for AGEs and their accumulation in diabetes and related complications has been well demonstrated and realized, the accumulation of AGEs and their specific role in the regulation of hypertension in diabetes is still unclear. The second aspect of our work was the effect of the AG treatment on the RAS. The alteration in the RAS under diabetes is well known. We have focused our efforts on the action of the most important pressor hormone of the RAS, angiotensin II, and the receptors that mediate the actions of Ang II, the AT_1_ and AT_2_ receptors.

The present study was thus aimed at addressing the existing gaps in RAS biology using an STZ-induced rat model of diabetes to investigate the role of AG in mitigating diabetic complications.

## Materials and methods

Chemicals

Streptozotocin (S0130) and aminoguanidine hydrochloric acid (CAS 396494) were purchased from Sigma-Aldrich (St. Louis, MO, USA). All other fine chemicals used in the study were purchased from Thermo Fisher Scientific (Waltham, MA, USA). The antibodies for AT_1_ and AT_2_ receptors (PA5-20812 and PA5-20813, respectively) were purchased from Thermo Fisher Scientific, and the β-actin antibody was obtained from Cell Signaling (catalog #3700S; Danvers, MA, USA). The Femto Chemiluminescence Substrate was purchased from G-Biosciences (St. Louis, MO, USA). All other chemicals used for immunoblotting were purchased from Bio-Rad (Hercules, CA, USA).

Animals

The experimental protocol was approved by the Institutional Animal Use and Care Committee of the University of Hyderabad (approval number: UH/IAEC/AHS/2021-22/07). Age-matched male Sprague-Dawley rats (10-11-week-old), weighing approximately 200-225 g, were procured from the Indian Council Medical Research Animal Facility at the National Institute of Nutrition, Hyderabad. The animals were housed at the University of Hyderabad Animal Facility and had free access to standard rat chow and tap water.

Experimental design

Animals were assigned to four groups (n = 4-6): Vehicle, Vehicle+AG, STZ-induced, and STZ-induced+AG rats. After the acclimatization period, a single intraperitoneal (IP) injection of STZ (55 mg/kg) dissolved in 0.5 mM sodium citrate buffer (pH 4.0) was used to induce type 1 diabetes, as described previously [11]. The animals in the control group (Vehicle) were injected with 0.5 mM sodium citrate buffer (pH 4.0). After 48 hours of the injection, glucose levels in the blood drawn from the tail vein were measured; animals having blood glucose levels > 300 mg/dL were included in this study. The blood glucose levels in the Vehicle rats were also determined to ensure that they had not developed diabetes. Rats in the STZ-induced and Vehicle groups were administered AG at a dose of 1 g/L in drinking water for two weeks after the confirmation of their non-diabetic and diabetic conditions.

Measurement of blood glucose levels

Blood glucose levels were measured using a OneTouch Select Plus Glucometer (ONECARE, Bengaluru, India).

Determination of body weight, and food and water intake

The body weight, and food and water intake were measured over the course of treatments and also after transferring the rats to metabolic cages for 2-3 days prior to their sacrifice. The food intake was measured by providing the rats in individual cages with a weighed amount of food and determining the weight of the leftover food in each cage on the next day. Similarly, water intake was measured by determining the average amount of water consumed by the rats from a calibrated water bottle.

Determination of renal functions

Renal function parameters were determined in partial modifications of the methods, as described previously [13]. Briefly, rats were transferred to metabolic cages for 2-3 days to collect urine samples for calculating the urine flow rate (UFR), glomerular filtration rate (GFR), and urinary sodium and protein levels [15]. A clinical biochemistry analyzer (Beckman Coulter and Olympus) was used to determine the levels of creatinine and electrolytes in the urine. The urinary protein levels were determined by the spectrophotometric method, using the bicinchoninic acid (BCA) method.

Calculation of GFR

The glomerular filtration rate in the various groups of rats was calculated using the following formula: GFR = urine creatinine × urine flow rate (µL/minute)/serum creatinine.

Calculation of urinary sodium excretion rate

The urinary sodium excretion rate, as an index of renal function and natriuresis, was calculated using the following formula: urinary sodium excretion rate = urine sodium concentration (mmol/L) × 24 hour-urine volume/mMoles of sodium.

Histopathological analysis of the kidney

For histopathological analysis, the kidneys from each animal were excised at the time of sacrifice and placed in 10% formalin, as described previously [24]. They were then dehydrated and embedded in paraffin. The paraffin-embedded kidney tissues were sectioned at a thickness of 5 µm and stained with hematoxylin and eosin (H&E). The sections were examined by a pathologist. At least 4-10 random fields were examined per section, comparing 10 or more glomeruli in each field.

Immunoblot analysis

The kidney was dissected from the sacrificed rats, as described previously [15]. The kidney homogenates were centrifuged at 14,000 rpm for 15 minutes at 4°C, and the supernatants were recovered. Total protein was quantitated using the BCA method by measuring the absorbance at 562 nm, according to the manufacturer’s protocol.

For immunoblot analysis, the supernatant of the kidney homogenate was dissolved in 4× loading sample buffer, containing β-mercaptoethanol, and boiled for five minutes at 95°C. The proteins (35 µg protein for AT_1_ and 40 µg protein for AT_2_) were separated using 10% sodium dodecyl sulfate-polyacrylamide gel electrophoresis (SDS-PAGE) and transferred onto a nitrocellulose membrane. The membrane was blocked with 5% bovine serum albumin in phosphate-buffered saline (PBS) with 0.1% Tween-20. The blots were incubated with the primary antibodies (polyclonal AT_1_ (1:1000) and AT_2_ (1:1000)) and subsequently with horseradish peroxidase (HRP)-conjugated anti-rabbit secondary antibody (1:5000). The signal was detected using a chemiluminescence reagent, and bands were visualized using the ChemiDoc XRS instrument (Bio-Rad). The blots were stripped off the antibodies and reprobed for β-actin (1:3000) (catalog #3700S; Cell Signaling) as a loading control. Densitometry analysis of the bands was performed using the ImageJ software (NIH, USA), and AT_1_ and AT_2_ levels relative to those of β-actin were determined for all the groups.

Statistical analysis

Results are expressed as mean ± standard error of the mean (SEM). Data were analyzed using GraphPad Prism 6. Student’s t-test was used to compare between the groups. A p-value < 0.05 was considered statistically significant.

## Results

AG treatment of STZ-induced diabetic rats prevented an increase in blood sugar levels and affected water and food intake and body weight

**Figure 1:**
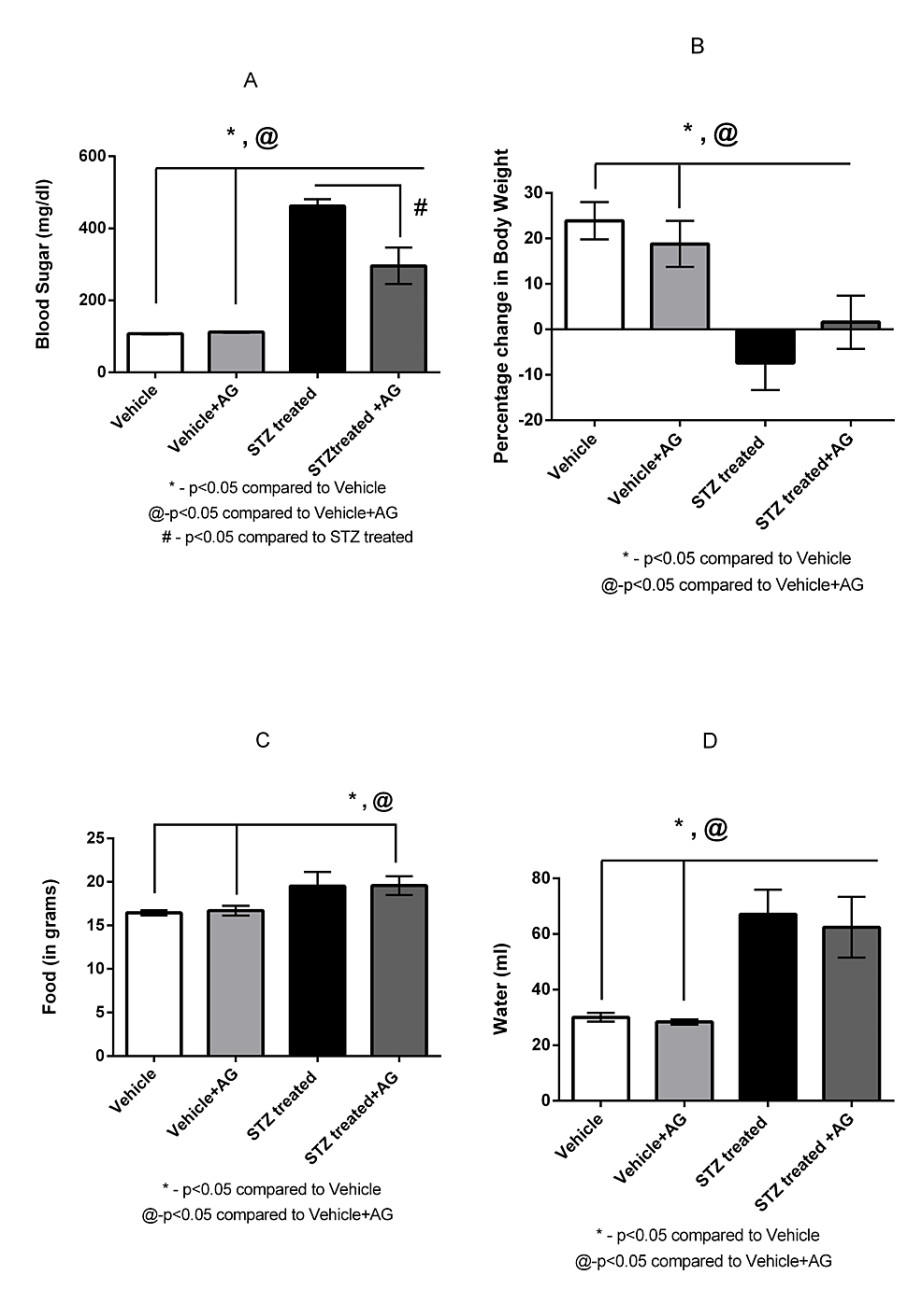
Effect of AG treatment on diabetic rats on blood sugar levels and hemodynamic patterns (n = 6-8) Effect of AG treatment (1 g/L) on blood glucose level (A), body weight (B), food consumption (C), and water consumption (D) in Vehicle and STZ-induced diabetic rats. Values are means ± SEM. *p < 0.05 compared to Vehicle rats, @p < 0.05 compared to Vehicle+AG rats, and #p < 0.05 compared to STZ-induced diabetic rats (Student’s t-test) AG: aminoguanidine, STZ: streptozotocin, SEM: standard error of the mean

Two weeks after the induction of diabetes, STZ-treated rats had significantly increased blood glucose levels, increased water consumption, and decreased percent change in body weight compared to the corresponding parameters in the Vehicle group (462.3 ± 18.6 versus 109.7 ± 1.25 mg/dL (p <0.05) (n = 8), 67.13 ± 8.78 versus 30.06 ± 1.56 mL (p < 0.05), and -7.35 ± 5.97 versus 23.87 ± 4.1 g (p < 0.05), respectively) (Figure 1A, 1B, 1D). A significant change in food consumption was also observed between rats in the STZ-induced diabetic and Vehicle groups (19.51 ± 1.61 versus 16.44 ± 0.29 g (p<0.05)) (Figure 1C).

The administration of AG (1 g/L) to the STZ-induced diabetic rats significantly decreased blood glucose levels, modestly decreased water consumption, had no difference in food consumption, and caused a slight restoration in the percent change in body weight in comparison with the STZ-treated rats (295.9 ± 50.69 versus 462.3 ± 18.6 mg/dL (p<0.05), 62.44 ± 10.93 versus 67.13 ± 8.7 mL, and 1.6. ± 5.8 versus -7.35 ± 5.97 g, respectively) (Figure 1A, 1B, 1D). No difference was recorded between food consumption in STZ-induced diabetic rats and STZ-induced diabetic rats treated with AG (19.51 ± 1.61 versus 19.58 ± 1.08 g) (Figure 1C). There was a significant change in blood sugar levels, and food and water consumption between the STZ-induced+AG rats and the Vehicle+AG rats (295.9 ± 50.69 versus 111.6 ± 1.49 mg/dL, 19.58 ± 1.08 versus 16.70 ± 0.5 g, and 62.44 ± 10.93 versus 28.40 ± 0.93 (p < 0.05)) (Figure 1A, 1B, 1D). A significant difference was also noted in the percent change in body weight between the STZ-induced+AG rats and the Vehicle+AG rats (1.6 ± 5.8 versus 18.8 ± 5.9 (p < 0.05)) (Figure 1B). No differences in these parameters were noted between the Vehicle and Vehicle+AG groups (blood sugar: 109.7 ± 1.25 ± 1.8 versus 111.6 ± 1.49 mg/dL, water intake: 30.06 ± 1.56 versus 28.40 ± 0.93 mL, percent change in body weight: 23.87 ± 4.1 versus 18.8 ± 5.9 g, food intake: 16.44 ± 0.3 versus 16.70 ± 0.5) (Figure 1A-1D).

STZ-induced diabetic rats had increased urinary protein levels, urine flow rate, GFR, and sodium excretion

**Figure 2:**
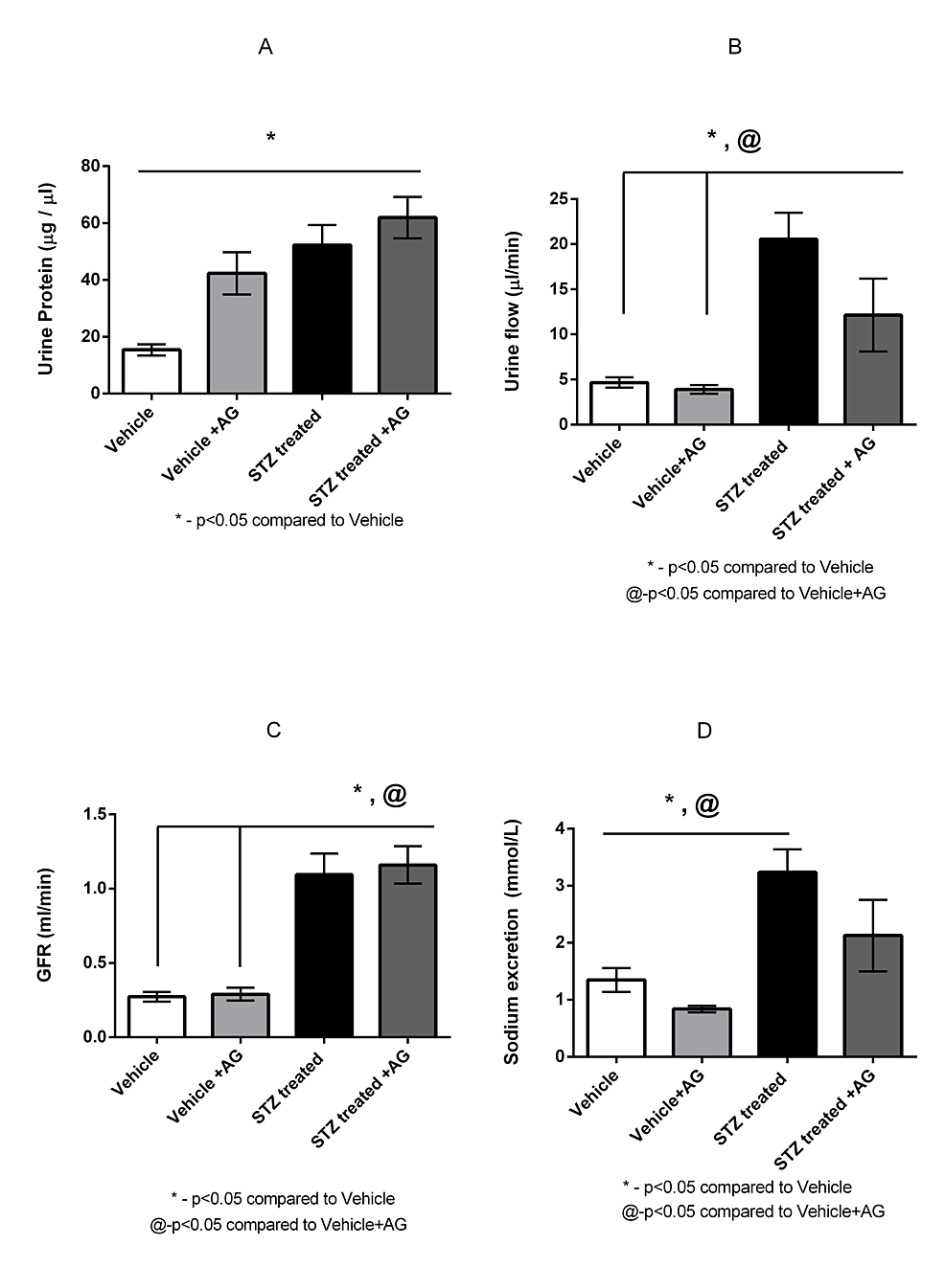
Effect of AG treatment on diabetic rats on physiological and renal functions (n = 3-5) Effect of AG treatment (1 g/L) on urinary protein level (A), urine flow rate (B), GFR (C), and urinary sodium level (D) in Vehicle and STZ-induced diabetic rats. Values are means ± SEM. *p < 0.05 compared to Vehicle rats and @p < 0.05 compared to Vehicle+AG rats (Student’s t-test) AG: aminoguanidine, GFR: glomerular filtration rate, STZ: streptozotocin, SEM: standard error of the mean

The diabetic rats had significantly higher urine protein levels and increased UFR, GFR, and urine sodium than the Vehicle group rats (52.23 ± 7.16 versus 15.14 ± 1.94 µg/µL (p < 0.05), 20.55 ± 1.94 versus 4.6 ± 0.57 µL/minute (p < 0.05), 1.1 ± 0.08 versus 0.27 ± 0.02 mL/minute (p < 0.05), and 3.24 ± 0.40 versus 1.35 ± 0.21 mmol/L (p < 0.05)) (Figure 2A-2D).

The administration of AG (1 g/L) slightly, albeit not significantly, increased the urine protein levels in STZ-induced+AG-treated rats compared with that in STZ-treated rats (61.95 ± 7.30 versus 52.23 ± 7.16 µg/µL). Surprisingly, the urine protein levels in Vehicle-treated rats that were administered AG were significantly increased compared with those in Vehicle-treated rats (42.35 ± 7.45 versus 15.14 ± 1.94 µg/µL (p < 0.05)). Urine protein levels were substantially increased in STZ-treated rats that were administered AG compared with those in Vehicle-treated rats that were administered AG, although not significantly (61.95 ± 7.30 versus 7.02 versus 42.35 ± 7.45 µg/µL) (Figure 2A). The administration of AG significantly decreased UFR (Figure 2B) in STZ-induced+AG rats compared with that in STZ-induced rats (12.14 ± 4.043 versus 20.55 ± 2.93 µL/minute (p < 0.05)). There was no effect on the UFR in Vehicle+AG and Vehicle rats (3.90 ± 0.48 versus 4.6 ± 0.57 µL/minute). However, the UFR was significantly increased in STZ-induced+AG rats than that in Vehicle+AG rats (12.14 ± 4.04 versus 3.90 ± 0.48 µL/minute (p < 0.05)) (Figure 2B).

AG administration did not affect the GFR in STZ-induced+AG rats compared with that in STZ-induced rats (1.10 ± 0.07 versus 1.1 ± 0.08 mL/minute) (Figure 2C), and the GFR was also similar in Vehicle+AG and Vehicle rats (0.29 ± 0.02 versus 0.27 ± 0.01 mL/minute). The GFR was, however, significantly increased in STZ-treated diabetic rats and STZ-induced+AG rats than in Vehicle rats and Vehicle+AG-treated rats (Figure 2C).

STZ-induced diabetic rats had significantly increased urine sodium levels than Vehicle rats (3.24 ± 0.40 versus 1.35 ± 0.21 mmol/L (p < 0.05)) (Figure 2D). The administration of AG resulted in considerably decreased urine sodium levels, although not significant, in the STZ-induced+AG rats compared with that in the STZ-induced rats (2.12 ± 0.63 versus 3.24 ± 0.40 mmol/L) (Figure 2D). The urine sodium levels in Vehicle+AG rats were similar to those in Vehicle rats (2.12 ± 0.06 versus 1.35 ± 0.21 mmol/L). However, the urine sodium levels in the STZ-induced+AG rats were increased compared with those in the Vehicle+AG rats (2.12 ± 0.63 versus 0.84 ± 0.06 mmol/L (p < 0.05)) (Figure 2D). Moreover, urine sodium levels were significantly increased in the STZ-induced diabetic rats compared with those in the Vehicle+AG rats (3.24 ± 0.40 versus 0.84 ± 0.06 mmol/L (p < 0.05)) (Figure 2D). No difference was recorded in the urinary sodium concentrations between the Vehicle rats and the Vehicle+AG-treated rats (1.35 ± 0.21 mmol/L versus 0.84 ± 0.06 mmol/L) (Figure 2D).

STZ-induced diabetic rats showed increased glomerular damage

**Figure 3:**
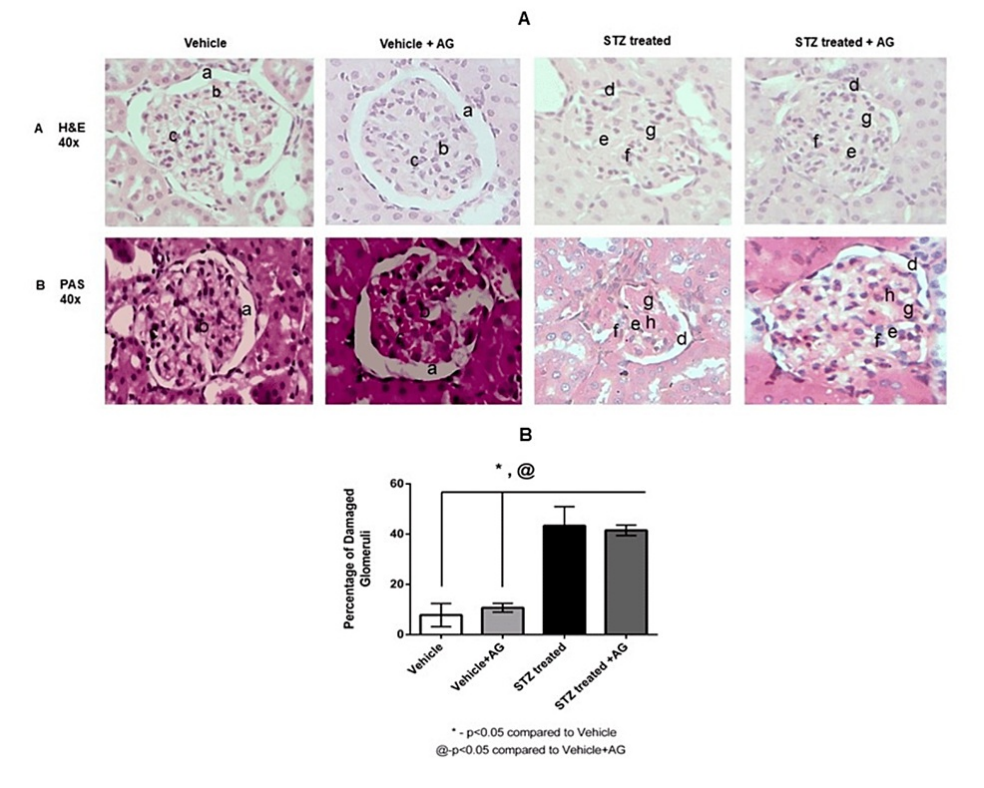
Histopathological changes in the kidney of diabetic rats and AG-treated rats (A) and glomerular damage expressed as percentage damage in diabetic rats and AG-treated rats (B) (n = 3-5) Effect of AG treatment (1 g/L) on renal damage as visualized with hematoxylin and eosin (H&E) (3A, Panel A) and PAS staining (3A, Panel B). Effect of AG treatment (1 g/L) on glomerular damage (B) in Vehicle and STZ-induced diabetic rats. Values are means ± SEM. *p < 0.05 compared to Vehicle rats and @p < 0.05 compared to Vehicle+AG rats (Student’s t-test) AG: aminoguanidine, H&E: hematoxylin and eosin, PAS: periodic acid-Schiff, STZ: streptozotocin, SEM: standard error of the mean

Significant changes were noted in the glomerular structures between the STZ-induced diabetic rats and the Vehicle rats (Figure 3A, Panel A and B); the extent of glomerular damage (%) was recorded (Figure 3B). While the Vehicle rats had a normal glomerulus showing normal Bowman’s space and capillaries, the STZ-induced rats showed obliteration of the Bowman’s space, collapsed capillaries, mesangial cell proliferation, and deposition of hyaline material (Figure 3A, Panel A and B). The STZ-induced diabetic rats and STZ-induced+AG-treated rats both showed diffused deposition of hyaline material with collapsed capillaries. These are the classical features of diabetic nephropathy that were visualized in the kidneys of diabetic rats using H&E staining. A greater deposition of periodic acid-Schiff (PAS)-positive material was observed in diabetic rats than in Vehicle rats. Mesangial cell proliferation was increased in diabetic rats (STZ and STZ-induced+AG) than in Vehicle rats (Vehicle and Vehicle+AG) (Figure 3A, Panel B). The extent of glomerular damage was significantly increased in STZ-induced diabetic rats than in Vehicle rats (43.34 ± 7.60 versus 7.79 ± 4.60 (p < 0.05)) (Figure 3B). The administration of AG (1 g/L) did not affect the changes in the glomerular damage, as observed for the STZ-induced diabetic rats (41.50% ± 2.06% versus 43.34% ± 7.60%). No significant difference in the glomerular damage was recorded between the Vehicle+AG rats and the Vehicle rats (10.67 ± 1.76 versus 7.79 ± 4.60). However, significantly increased glomerular damage was observed in STZ-induced+AG rats than in Vehicle+AG rats (41.50% ± 2.06% versus 10.67% ± 1.76% (p < 0.05)).

STZ-induced diabetic rats had increased expression of Ang II and type 1 (AT_1_)_ _and type 2 (AT_2_) receptors

**Figure 4:**
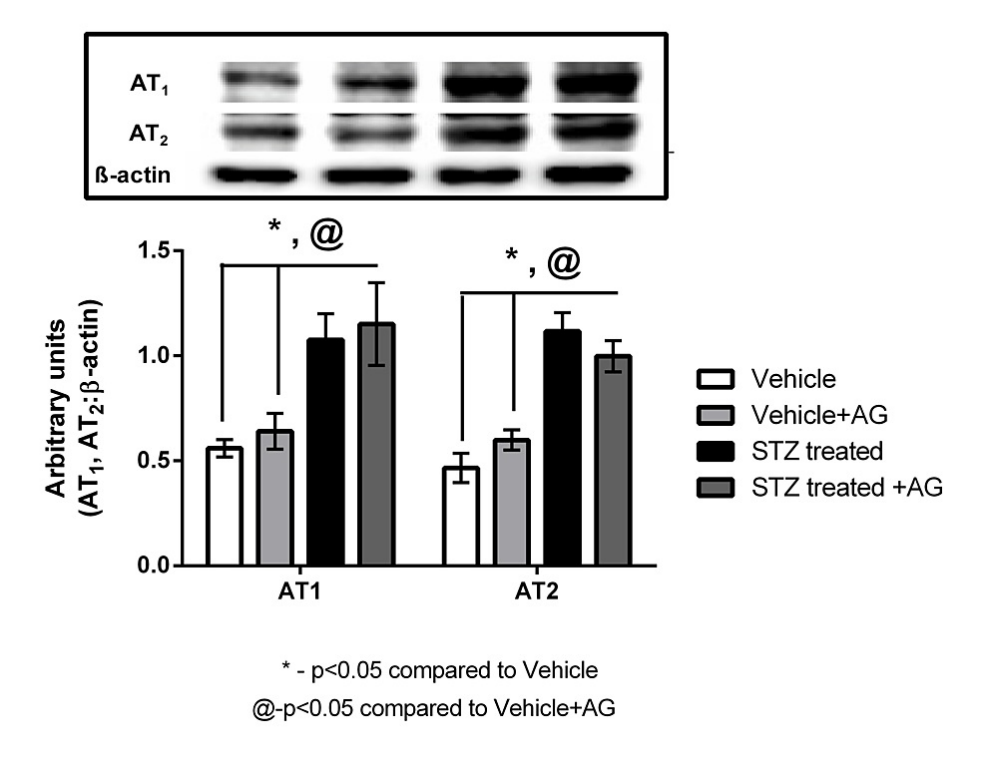
Angiotensin II, and type 1 and type 2 receptor expressions (AT1 and AT2) in the kidneys of diabetic and AG-treated rats (n = 3-5) Effect of AG treatment (1 g/L) on the expression of AT_1_ and AT_2_ receptors in the kidney of the Vehicle and STZ-induced diabetic rats, normalized against β-actin. Values are means ± SEM. *p < 0.05 compared to Vehicle rats and @p < 0.05 compared to Vehicle+AG rats (Student’s t-test) AG: aminoguanidine, STZ: streptozotocin, SEM: standard error of the mean

The expression of the AT_1_ and AT_2_ receptor proteins in the kidney tissue was determined using western blot analysis. The AT_1_ and AT_2_ receptor antibodies detected a band of approximately 45 kDa. Densitometry analysis showed that the levels of the AT_1_ receptors were significantly increased in STZ-induced rats compared with those in Vehicle rats (1.08 ± 0.12 versus 0.55 ± 0.04, 1.9-fold (p < 0.05)) (Figure 4). AT_2_ receptor levels were also significantly increased by almost 2.6-fold in the STZ-induced diabetic rats compared with those in the Vehicle rats (1.12 ± 0.08 versus 0.46 ± 0.07 (p < 0.05)) (Figure 4). However, upon treatment with AG, the STZ-induced+AG rats showed a modest decrease (0.99 ± 0.07 versus 1.12 ± 0.08) in AT_2_ receptor levels (Figure 4). On the contrary, AT_1_ receptor levels did not change in the STZ-induced+AG rats compared with those in the STZ-induced diabetic rats (1.1 ± 0.19 versus 1.08 ± 0.12) (Figure 4).

## Discussion

In the present study, we identify that after two weeks of STZ administration, (i) there was a significant increase in blood glucose levels in the STZ-treated rats, (ii) AG treatment prevented the increase in the blood glucose levels in the STZ-induced+AG group, compared with that in STZ-induced diabetic rats, (iii) there was a significant increase in the urinary sodium levels in the STZ-induced diabetic rats, (iv) considerable renal changes, characteristic of diabetic lesions, were present in the diabetic rats, and (v) the levels of Ang II receptors, AT_1_ and AT_2_, were significantly increased in the diabetic group of animals; the increase in the AT_2_ receptor levels was more compared with that in the AT_1_ receptor.

The increase in blood sugar levels in the STZ-induced diabetic rats was accompanied by a decrease in body weight and an increase in water intake compared with that in the Vehicle rats. The observed changes in these parameters, which are established phenotypes of diabetes, suggest that we could develop a good animal model of diabetes.

The AG treatment of the STZ-induced diabetic rats prevented the increase in blood glucose levels compared with that in untreated STZ-induced diabetic rats. The AG treatment was also helpful in restoring the weight loss vis-à-vis the STZ-induced diabetic rats (Figure 1A-1C). The most plausible explanation for this could be the fact that AG prevents the formation of AGEs by reacting with sugar [18].

Additionally, the inhibitor treatment partially or modestly reversed the conditions as observed in the STZ-induced diabetic rats, which included decreased UFR and improved body weight (Figure 1B, 2B). However, AG did not affect a few other parameters, including renal damage (Figure 3A, 3B). An anomalous or selective action of AG was thus demonstrated in this study.

We observed an increased expression of the AT_1_ and AT_2_ receptors in the kidneys; this is in consonance with previous results (Figure 4). It is important to note that the fold increases in the AT_2_ receptors in the STZ-induced diabetic rats are more (2.6) compared with that of the AT_1_ receptors (1.9). The levels of AT_1_ receptors have also been reported to be significantly increased in obese Zucker rats, a model of hyperinsulinemia, and to stimulate the Na+/K+ ATPase pump [25]. It is, therefore, tempting to comment on the anti-natriuretic and natriuretic functions of AT_1_ and AT_2_, respectively, which regulate the sodium levels in the body. We also believe that the natriuretic effects of the AT_2_ receptors overcome the anti-natriuretic effects of the AT_1_ receptors. This is a very common feature in early-stage diabetes where hyperfiltration could lead to increased sodium excretion.

The increased urinary sodium levels in the diabetic rats (Figure 2D) could be attributable to the increased AT_2_ receptor levels in the diabetic rats, as reported previously [14,15]. The cGMP/NO pathway has been shown to be responsible for the inhibition of the sodium pump and causes increased excretion of sodium from the body. We have also obtained similar results and are, therefore, highly inclined to suggest that the increased sodium excretion in the present model is mediated through similar mechanisms [26]. We also demonstrated increased sodium excretion in STZ-induced diabetic rats, which implies an increase in natriuretic functions in the STZ-induced diabetic rats (Figure 2D). STZ-treated rats excreted higher volumes of urine and also had a higher UFR (Figure 2B) and thus have a higher GFR in comparison with Vehicle rats (Figure 2C). These are characteristics of diabetic complications, as demonstrated in early diabetic conditions.

We observed renal injuries, namely, collapsed capillaries in the Bowman’s capsule, mesangial cell proliferation, and deposition of the hyaline material, in STZ-treated rats; such injuries are characteristics of diabetic lesions (Figure 3A, Panel A and B). This is also manifested by increased protein levels in the urine due to functional changes in the nephron (Figure 2A). Surprisingly, the Vehicle+AG rats also showed slightly increased urine protein levels than the Vehicle rats, which may be attributable to an acute effect, most likely induced by AG (Figure 2A). However, we did not see any noticeable changes in the glomerular structure, which implies an acute effect, causing proteinuria, probably affecting the tubular structure. This notion is apparently more credible considering the fact that the urinary protein levels were also enhanced in the STZ-induced+AG rats than in the STZ-induced rats. This is more likely an acute phenomenon that is induced by AG treatment, which warrants further investigation.

The histopathological examination of kidney glomerular sections from STZ-induced diabetic rats showed obliteration and narrowing of the capillary space within the Bowman’s capsule, hypercellularity of the glomerulus, occlusion, and collapsed capillary lumens (Figure 3A, Panel A and B). We noted an increased intensity of PAS positivity in glomeruli and basement membrane thickening, which indicates the existence and progression of diabetic complications (Figure 3A, Panel B). This may be due to the severe and sustained hyperglycemia in these animals, as reported in several studies [27].

In addition, the levels of AT_1_ and AT_2_ were significantly increased in STZ-induced rats (Figure 4); this could be due to increased blood glucose levels affecting renal function by activating the RAS, as observed in previous studies [28].

AG administration partially or moderately reversed polydipsia and food intake in STZ-induced diabetic rats (Figure 1C, 1D) and, thus, affected the physiological and metabolic parameters.

Although AG administration did not ameliorate the renal injury in STZ-induced rats, it did cause significant reversion of the UFR and did not increase the GFR further (Figure 2B, 2C), implying a role in the protection of renal functions. This is because despite the prevention of the increase in blood glucose levels, the levels were still high enough in the STZ-induced+AG rats (Figure 1A) for causing the observed complications. This indicates that the AG treatment improved renal functions by preventing an increase in blood glucose levels. However, AG administration did not prevent glomerular damage (Figure 3A, Panels A and B, and Figure 3B). The functions of AT_1_ and AT_2_ receptors have always attracted a lot of attention. However, the effect of AG on their functions, in context of the sodium metabolism, is yet to be explored fully. The role of the AT_1_ receptors in the stimulation of Na+/K+ ATPase in the proximal tubule of STZ-induced diabetic rats has been suggested [11].

Additionally, the AT_2_ receptor was shown to play an important role in renal sodium excretion in obese Zucker rats and in STZ-induced diabetic rats using AT_1 _and AT_2_ receptor antagonists [14,15].

In the present study, the levels of both AT_1_ and AT_2_ receptors were increased in STZ-induced diabetic rats (Figure 4). However, the enhanced sodium excretion in the STZ-induced diabetic rats compared with that in the Vehicle rats indicates that the AT_2_ receptor dominates the anti-natriuretic function of the AT_1_ receptor via an increased inhibitory effect of the Na+/K+ ATPase activity in proximal tubules. These results are in agreement with those reported previously [14,15].

AG administration led to a considerably decreased sodium excretion in the STZ-induced diabetic rats. This finding correlates with the renal levels of the AT_2_ receptor after AG administration, wherein we noted decreased levels of the AT_2_ receptor. We did not observe any change in the renal levels of the AT_1_ receptor after AG administration, which indicates that there is no major difference in the anti-natriuretic function attributed to the Na+/K+ ATPase activity in the kidney, between these groups of diabetic rats. We, therefore, speculate that after AG administration, decreased levels of AT_2_ receptors in the kidney, in comparison with those in the STZ-induced diabetic rats, may not have a major role in regulating sodium homeostasis, as is the case with the STZ-induced diabetic rats. We postulate here that in diabetes, due to a lack of insulin, there is a decreased sodium re-absorption by the kidney. A decreased sodium level in the body triggers the release of renin as a compensatory mechanism to restore sodium balance. Moreover, insulin levels affect the AT_1_ receptor function [10], and hence, we believe that in the STZ+AG-treated rats, due to a prevention of the increase in blood glucose levels, assuming mainly due to increased insulin sensitivity, helping in the better utilization of glucose, we see an increased anti-natriuretic function of the AT_1 _receptors, compared to the increased natriuretic function of the AT_2_ receptors in the STZ rats.

The findings of the present study indicate a selective and differential effect of the AG treatment on diabetic rats. Further studies are warranted to understand the long-term effects of AG inhibitors on diabetic rats. The estimation of the time and dose of STZ to be employed that can cause the disease and elicit all the complications of diabetes will be challenging. The early diabetic condition might not be associated with AGEs; hence, induction of diabetes and subsequent accumulation of AGEs require extensive time-dependent studies. Because no particular time frame can be guessed for such accumulation to occur, this investigation needs a more focused time-dependent study to identify the benefits of AG treatment in diabetic rats. In physiological studies, conditions, factors, and homeostatic balance vary from animal to animal, and achieving statistically reliable numbers for a desired parameter sometimes becomes difficult.

The animal model that we used is a very good model to study diabetic complications. This is owing to the important ramifications of diabetes that we have observed and are mimicked in our model with regard to the clinical phenotypes, physiological determinants, and renal function. The breakdown of AGEs is being utilized in treating hypertensive conditions in diabetes [29]. With regard to the prevention of an increase in blood glucose levels in the presence of AGEIs, our study adds newer dimensions to understanding the role of AGEs in diabetes. This study highlights the potential of AG for therapeutic application in the management of diabetes.

## Conclusions

We postulated that the prevention of AGE accumulation or abrogation of AGE synthesis by using an AGE inhibitor, aminoguanidine (AG), in streptozotocin (STZ)-induced diabetic animal models, would affect the progression of diabetes and its related complications. We present the following major and interesting findings in this manuscript: (i) treatment with AG prevented an increase in blood sugar levels in STZ+AG-treated rats compared to STZ-treated rats and (ii) the expression of Ang II type 2 receptor, AT_2_, is significantly increased, facilitating increased natriuresis in the diabetic rats. Increased natriuresis, as mediated by the Ang II type 2 receptor, AT_2_, thus serves as an important regulator of sodium levels in the body and hence blood pressure in diabetic conditions. These studies indicate new dimensions of the AG treatment, where it could have a protective effect in preventing or delaying the deleterious effects of diabetes. Further studies are warranted to investigate further the efficacy of the inhibitor aminoguanidine (AG) in ameliorating diabetic complications.
